# Reduce stress and the risk of burnout by using yoga techniques. Pilot study

**DOI:** 10.3389/fpubh.2024.1370399

**Published:** 2024-04-02

**Authors:** Agnieszka Zok, Monika Matecka, Artur Bienkowski, Magdalena Ciesla

**Affiliations:** ^1^Department of Philosophy of Medicine and Bioethics, Poznan University of Medical Sciences, Poznan, Poland; ^2^Department of Occupational Therapy, Poznan University of Medical Sciences, Poznań, Poland

**Keywords:** yoga, stress, occupational stress, burnout, wellbeing

## Abstract

**Introduction:**

This article examines the effectiveness of yoga in managing stress, with a particular focus on work-related stress. Yoga combines physical postures, breath control, and meditation, and has gained recognition for its potential to relieve stress.

**Purpose:**

This study aimed to investigate the motivating factors behind individuals adopting yoga exercises and to assess the effects of regular yoga practice, with a particular focus on age-related differences. Additionally, we aimed to compare participants’ expectations with the actual results of their yoga practice.

**Methods:**

To achieve this, we conducted a comprehensive survey using an online form, which was completed by 520 yoga practitioners. Participants were surveyed about their motivation, the effects they experienced, and the type of yoga they practiced.

**Results:**

The results showed that the most common motivation for individuals practicing yoga was stress reduction. Additionally, the analysis of the effects of regular yoga practice demonstrated a significant reduction in stress levels, with experienced practitioners reporting lower stress levels compared to beginners. In conclusion, the study suggests that regular yoga practice can be an effective way to reduce stress levels.

**Conclusion:**

Dynamic forms of yoga, which incorporate fluid movements and synchronized breathing techniques, are highly effective approaches to stress management and relief. These findings highlight the value of yoga as a tool for individuals of all ages seeking stress relief and overall well-being. Another advantage of yoga practice is its affordability and lack of negative side effects.

## Introduction

In modern society, individuals are exposed to numerous stressors. In the European Region, approximately 50% of workers consider stress as a common occurrence. Occupational stress is a chronic condition that cannot be cured with a single treatment. To address this condition, it’s essential to understand its history or epidemiology. Once you have a clear understanding of the problem, you can explore various alternatives for protection, prevention, and intervention (1). Occupational stress is one of the most common work-related health problems worldwide (1–7). Occupational stress is a well-documented issue that poses risks to both physical and mental health. According to the World Health Organization (WHO), stress-related diseases are the leading cause of premature death in European countries. Work-related stress incurs high costs for workers, employers, and society. It is important to address this issue to promote a healthier and more productive workforce (8). Occupational stress is a significant modern health and safety challenge. The causes of occupational stress can be numerous, ranging from the fear of losing one’s job, to job burnout, lack of support, and mobbing. Whatever the cause, occupational stress not only significantly reduces the quality of life (9, 10) and leads to various diseases and disorders (10, 11), including cancer (12–14). An association between occupational stress and sleep quality has been also documented (15–18) A study involving a substantial cohort of firefighters revealed a correlation between heightened occupational stress and increased global suicide risk, lifetime suicide threats, and current suicidal intent. Furthermore, these associations were found to be diminished as self-reported distress tolerance increased (19).

Occupational stress can result in types of stress: behavioral (e.g., absenteeism), physical (e.g., headaches), and psychological (e.g., depressed mood) (20). Furthermore, this stress is associated with constant staying ready (fight-or-flight response) (21, 22). This can result in a variety of disorders, such as mental disorders (e.g., depression, anxiety, post-traumatic stress disorder), job dissatisfaction, maladaptive behavior (e.g., substance abuse), cardiovascular disease, and musculoskeletal disorders (23).

A landmark study by the World Health Organization and the International Labor Organization found that exposure to long working hours, theorized to result from increased psychosocial stress at work, is the occupational risk factor with the highest burden of disease, officially estimated to have caused the deaths of some 745,000 workers from ischaemic heart disease and stroke in 2016 (24). Work-related stress is also a problem of work-life balance, as shown by the Work, Family, and Health Study (25). Although the problem of work-related stress is not a new issue, the instability of today’s labor and employment market exacerbates it. Studies have shown that occupational stress is a cause of depression and anxiety states (26, 27). In addition, chronic stress significantly reduces the quality and productivity of work. Above all, however, it should be remembered that physiological responses to stress can accurately reflect health (28–30). Stress-related disorders are a group of increasingly diagnosed diseases worldwide (30–32). Unfortunately, conventional pharmacotherapy treatments are not always feasible (33–35). Other activities that positively impact the patient’s well-being can also support conventional therapy. Physical activity, including yoga, is a frequently recommended form of welfare support. Yoga is a unique form of physical activity because of the multifaceted nature of the practice. There is evidence that yoga has mood-enhancing properties, likely related to its inhibitory effects on physiological stress and inflammation, often associated with affective disorders (13, 36, 37). Yoga practice has already been described as an effective and safe intervention for people with depression, anxiety disorders, or PTSD (38–40). These findings have provided valuable insights into the potential benefits of yoga in managing these conditions. However, despite the existing evidence, there remains a need to explore the motivations and effects of yoga practice more comprehensively. Previous research suggests that health concerns, improved wellbeing, and stress reduction are the main motivations for starting a yoga practice (41–43). The British study also showed that the practice of yoga is used as a form of physical activity to complement the rehabilitation process (42).

The aim of this study was to investigate the motivations and effects of yoga practice among a group of randomly selected Polish yogis. The aim was to explore the role of yoga in reducing stress and improving overall well-being by understanding the perspectives and experiences of these practitioners. Specifically, the study analyzed survey responses from individuals who practice yoga with the explicit aim of reducing stress. Our research has the potential to provide valuable insights that can guide healthcare professionals and individuals seeking alternative approaches to stress management and mental health improvement.

## Materials and methods

The study was approved by the Bioethics Committee at PUMS 391/20.

### Participants

Yoga practitioners from different regions of Poland took part in this study. Participants were recruited from the yoga community through popular social media platforms such as Facebook and Instagram. A wide range of yoga styles were represented among the participants. The selection of these specific social media platforms aimed to include individuals within a specific age range.

The primary criterion for participation in the study was maintaining a consistent and regular yoga practice. Individuals who did not maintain a regular practice were excluded from the analyses. Table 1 shows the basic characteristics of these respondents, including their age, level of education, and place of residence. By completing the questionnaire, respondents explicitly confirmed their informed consent to participate in the study.

**Table 1:** The socio-demographic characteristics of polish yogis based on the results of a survey question and answers, including the number of respondents (N) and the corresponding percentage distribution (%).

Question	Variant of answer	*N* (%)
Gender	Female	*N* = 296, (89.16%)
	Male	*N* = 36, (10.84%)
Age	14–17	*N* = 1, (0.3%)
	18–24	*N* = 18, (5.42%)
	25–35	*N* = 54, (15.96%)
	36–46	*N* = 86, (25.3%)
	47–57	*N* = 96, (28.92%)
	58–69	*N* = 64, (18.98%)
	70–80	*N* = 12, (3.61%)
	81–90	*N* = 1, (0.3%)
Education	Higher education	*N* = 217, (65.36%)
	Primary education	*N* = 5, (1.51%)
	Secondary education	*N* = 95, (28.61%)
	Vocational	*N* = 15, (4.52%)
Residence	A city of over 100,000.	*N* = 160, (48.19%)
	City of 10–25 thousand.	*N* = 36, (10.84%)
	City under 10,000.	*N* = 22, (6.63%)
	City up to 100,000.	*N* = 61, (18.37%)
	Village	*N* = 53, (15.96%)

### Questionary form

The survey was created using Google Forms and distributed to participants through a link on leading yoga-focused websites. The form consisted of two parts. The first part was about the experience of practicing yoga, and the second was a metric.

The survey design allowed at most 5 min to complete. The questions sought answers to issues related to motivation to practice. In addition, the practice results were checked to see if they reflected the motivations.

### Statistic

The filled questionnaires were downloaded and saved in “xlsx” format. Statistical analysis and visualization of the collected data were conducted in the statistical software environment “R” (version 4.1.2), utilizing additional libraries. The xlsx files were imported into R using the “openxlsx” library (44). The number of response options given to the respective questions and the corresponding percentages were calculated. The resulting figures were displayed in tables or visualized using the ggplot2 library (45).

### Analysis of multiple-answer questions

The question regarding expectations of yoga practice was in the form of a multiple-choice question. The responses were segregated into distinct age categories (<18–24, 25–30, 31–35, 36–40, 41–45, 46–50, 56–60, 61–65>) to exhibit changes in expectations according to the age of the participants. The total number of responses, percentage per response, and percentage per responders population were calculated using the “multiResponse” function from the “user-friendly science” package (46).

To identify the trend of changes in expectations toward yoga with the age of respondents, pre-calculated values of percentage per response were used and a regression curve was calculated using a linear model. In addition, beta values, which quantify how strongly changes from one age category to the subsequent one affects the percentage response to a specific expectation, and R2 values, which measure the goodness of fit to the regression line, were obtained and presented in relevant figures.

### Cluster analysis

The multiple-choice questions were analyzed using the Ward hierarchical clustering method (47). Firstly, the answers were transformed into a binary matrix. Each column represented one possible answer option, and each row represented the answers given by one respondent. Next, the optimal number of clusters was determined by repeatedly calculating the sum of squared error (SSE) measurement with an increasing number of clusters. The result of dividing respondents’ answers into different clusters was presented as a heatmap, which was generated using the “Complexheatmap” package (48). Next, the selected questions were analyzed by dividing the respondents into groups obtained by assigning them to the corresponding clusters. The results with the percentage distribution were presented on bar charts.

The analysis of the relationship between motivation for practicing yoga and the effects of yoga practice.

The responses to the question about motivation for practicing yoga were grouped into subsets based on the frequency of each response. Next, in each subset, the number of responses and the percentage per response was calculated concerning the effects of yoga practice. Finally, the results were presented as a heatmap in which the percentage per response values were shown, assuming that the sum of the percentage per response equals 100 for each row (motivation).

## Results

The survey included 520 yoga practitioners. Women predominated among the respondents, accounting for 93.65% (487 people), while men accounted for 6.15% (32 people). One person declared himself non-binary without indicating his gender. Yogis were asked which style of yoga they most often practice. The vast majority of people, 41.6%, practice vinyasa yoga; 22.12% said they practice Ashtanga Yoga; however, the Iyengar method is practiced by 18.85%. 6.54 people declared the practice of spine and yin yoga by 5%. Another style than those mentioned is practiced by 5.96% of respondents. Regarding the place of practice, almost half of the respondents (49.62%) choose to practice at a yoga studio. 19.81% practice independently at home, while 16.15% use online instructors. 9.42% choose gyms. 5% of respondents began practicing during the covid-19 pandemic. We asked respondents about internship placement (the duration of their practice). Those practicing yoga for less than a year prevailed, accounting for 23.85% of the group. From 1 to 3 years, 38.27% of respondents have been practicing. Those practicing for over 10 years accounted for 12.5% of the group. The largest group, 35%, were yogis aged 36–45. Those aged 25–35 made up 31% of those surveyed. Residents of large cities with higher education predominated among those surveyed (Table 1).

**Figure 1:**
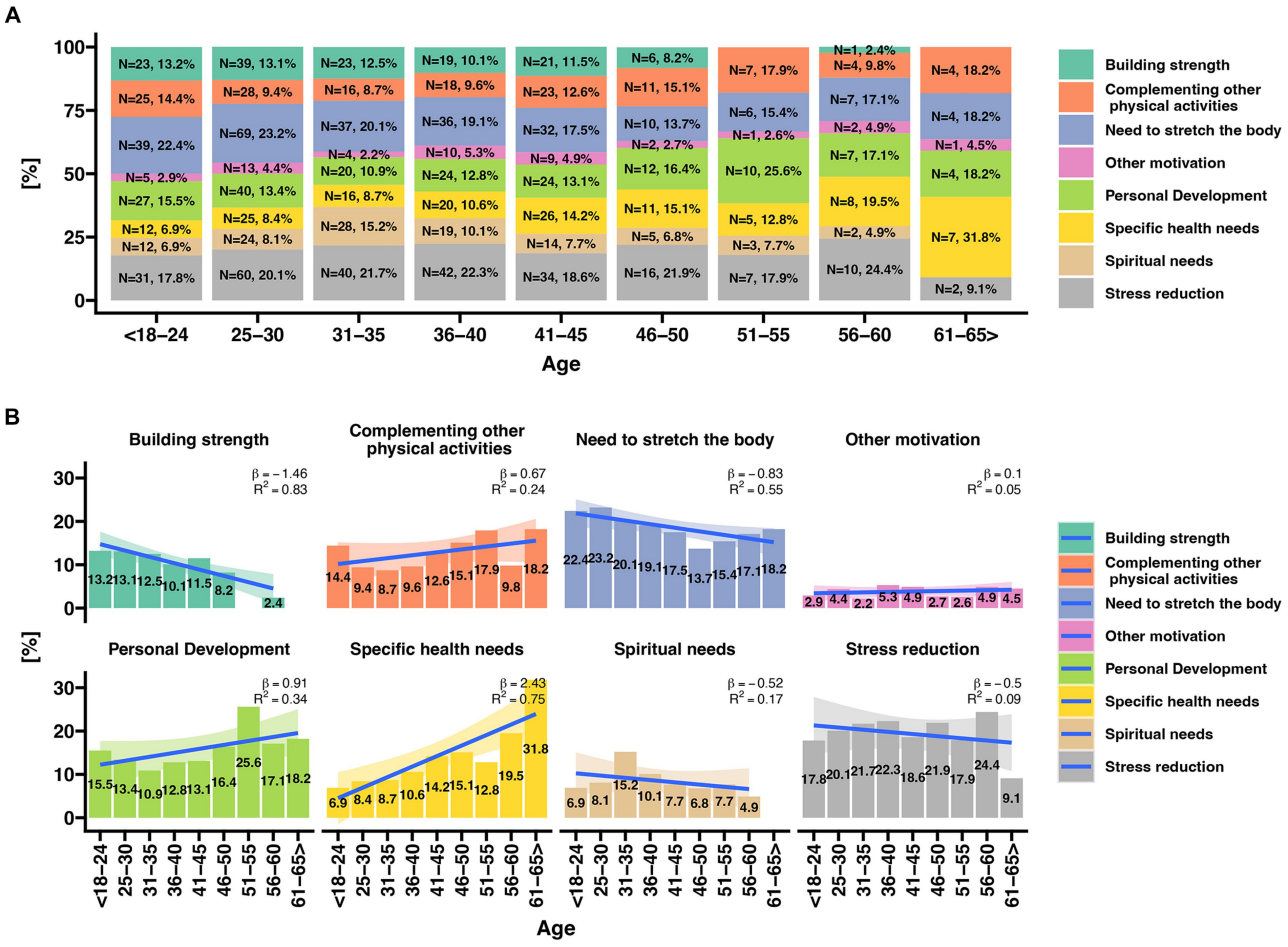
Analysis of motivation for practicing yoga in relation to the age of respondents. **(A)** Percentage distribution of responses [%] regarding motivation for practicing yoga in different age categories. **(B)** Analysis of changes in the frequency of specific responses to the question concerning motivation for practicing yoga in different age categories. The chart displays a regression line, including the beta value (indicating the magnitude of change during the transition from one age category to another) and R2 - the degree of fit of the data to the linear regression line.

Correlations were calculated between respondents’ stated expectations of yoga practice and their age. Among the motivating factors, stress reduction, body stretching, and personal growth were predominant. As yogis advanced in age, the frequency of citing specific health needs as a motivation for their practice increased. On the contrary, the need to strengthen the body decreases with age. However, the inclination to seek stress reduction through yoga remained consistent across all age groups except for those over 60. Individuals in this age group, more so than younger yogis, reported practicing yoga primarily due to specific health needs (Figure 1).

**Figure 2:**
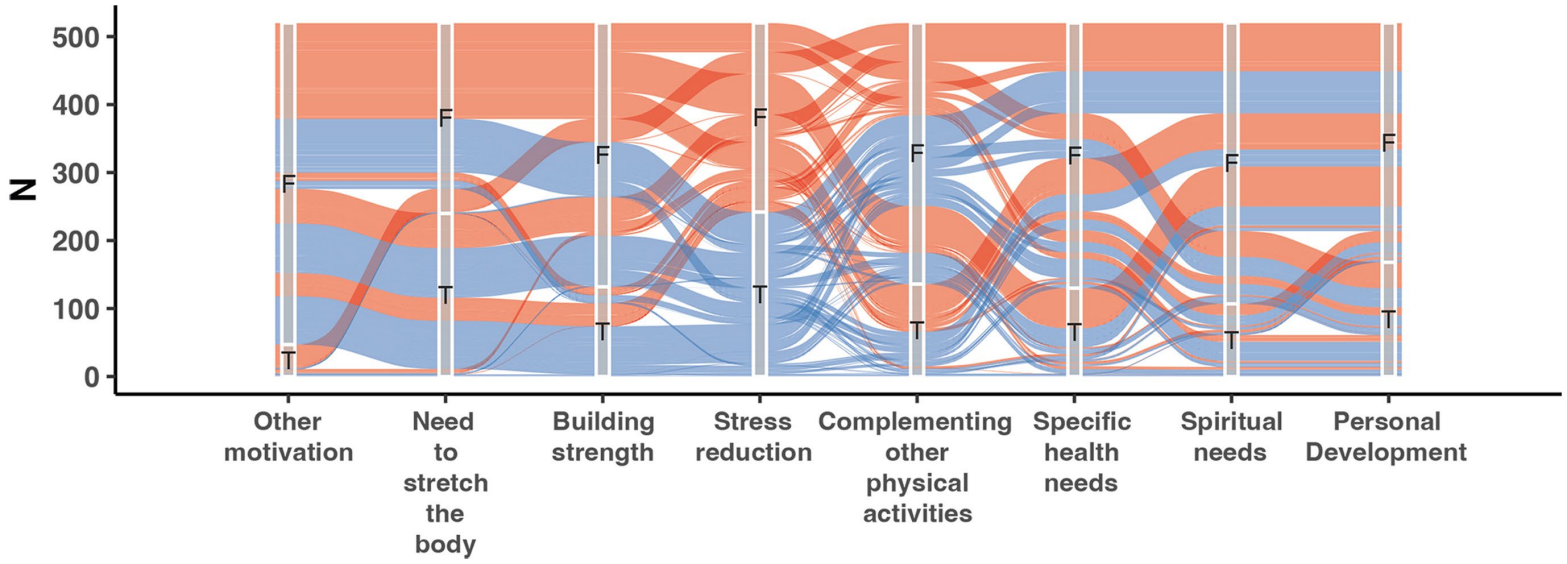
Alluvial Plot depending on the response indicating motivation for taking up yoga practice as stress reduction (blue lines, T- true) and in the case when respondents indicated motivations other than stress reduction (red lines, F-false). N - number of respondents. The x-axis represents all possible answer choices regarding motivation for taking up yoga practice.

Multiple-choice question answers regarding motivation to take up yoga practice in relation to stress reduction were preliminarily analyzed and visualized using alluvial plot (Figure 2). The chart depicts the number of respondents whose motivation for practicing yoga included stress reduction (T, blue color), as well as those who did not indicate stress reduction as one of the possible answers (N, red color). Approximately 50% of all participants indicated that their motivation for taking up yoga practice was stress reduction. Some of these individuals also selected the need for building strength and the need to stretch the body.

**Figure 3:**
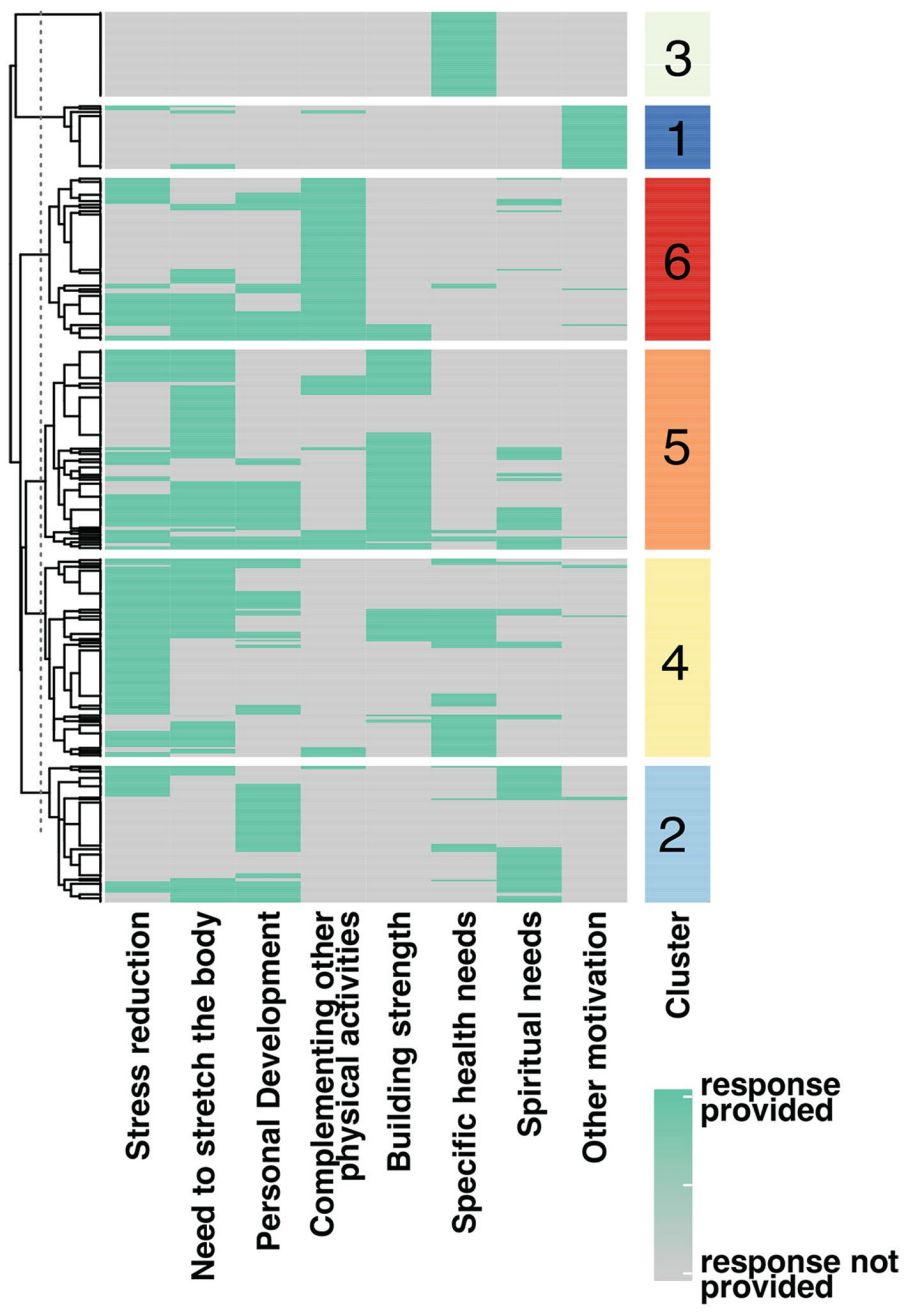
Cluster analysis based on multiple-choice questions related to motivation to take up yoga practice. The number of clusters is marked according to the color scale on the right side. The results of each survey respondent are presented in rows. The green color on the heatmap indicates the selection of a specific answer by the survey respondent.

Next, a detailed analysis was conducted to investigate the motivation behind taking up yoga practice. Prior to the analysis, respondents were divided into appropriate subgroups using the Ward hierarchical clustering method. Data analysis involved the repeated calculation of the sum of squared errors (SSE) to determine the optimal clusterization. The results indicated that dividing the data into six clusters, as shown in Figure 3, provided the most optimal clusterization. Each respondent was then assigned to their respective cluster. Respondents assigned to the first cluster mostly practiced yoga due to “other motivations” not mentioned in the analyzed question. Individuals classified into the second cluster primarily chose the responses “need for personal growth” and “spiritual needs”. The third cluster consists mainly of people practicing yoga for specific health needs. The fourth cluster was assigned to people expecting stress reduction and body stretching. Those assigned to the fifth cluster practiced stretching the body and building strength. In this cluster, yogis also declared a desire for personal growth and stress reduction. Finally, those supplementing other activities with yoga were assigned to cluster 6.

## A detailed analysis of responses regarding style, age, practice duration, and yoga values among survey participants assigned to respective clusters

### Style of yoga practice

**Figure 4:**
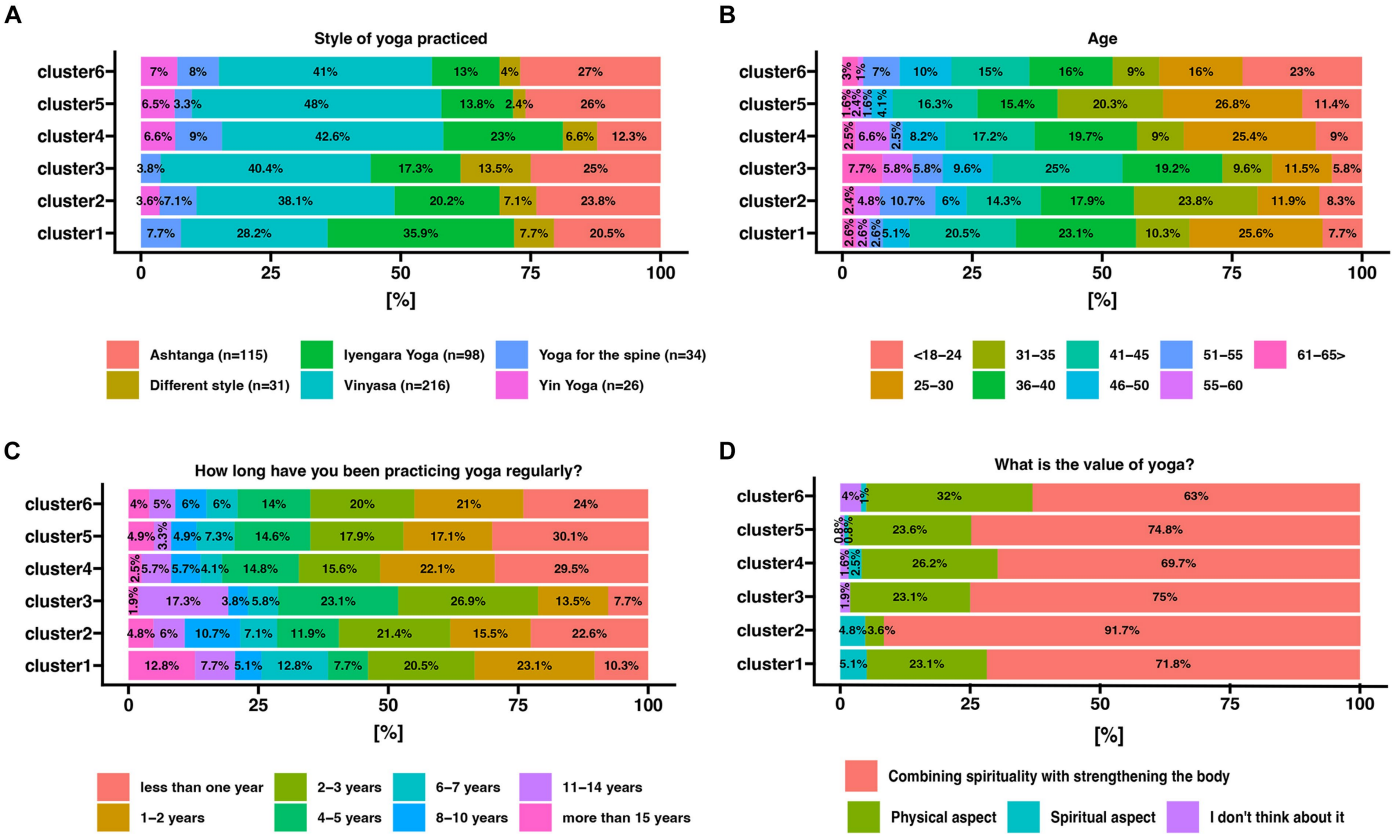
Comparison of response frequencies based on assignment to respective clusters. The percentage values of each response declaration are indicated on the x-axis, divided according to the previous assignment of yogis to their respective clusters. The color represents one of the possible answers to the given question, with their corresponding values presented in the legend. **(A)** Style of yoga practiced by participants classified into clusters; **(B)** Age of participants assigned to clusters; **(B)** Length of practice; **(C)** Significant values for yoga practitioners described according to clusters.

Participants whose motivation to practice yoga is mainly stress reduction (cluster 4) mainly practice the Vinyasa Yoga style. Compared to the other groups, those aiming to reduce stress also practiced Yin yoga and Spinal yoga. Those belonging to cluster 4 practiced Ashtanga yoga significantly less often (12.3%) concerning participants classified in the other clusters (20.5–27%) (Figure 4A).

### Age of yoga practitioners

Cluster 4 was dominated by people between 25 and 60 years of age. Yogis over 60 years of age predominated in cluster 3 in which the main motivation for undertaking yoga practice was based on specific health needs. The youngest participants in the study are most represented in cluster 6 in which they mainly see yoga as a complement to other sports (Figure 4B).

### Length of regular yoga practice

It was noted that the motivation for stress reduction was predominantly expressed by practitioners with short-term experience (those practicing for no more than 2 years accounted for over 51% in cluster 4). Yogis with many years of training were much more likely to attribute their practice to specific health needs or other motivations (Figure 4C).

### Value of yoga practice

We asked respondents which aspect of yoga practice they valued the most. For the vast majority, combining physical practice with spiritual elements is important. Many also value yoga as a physical activity The fewest number of people attend classes because of the spiritual aspects (Figure 4D).

### Does the motivation for practicing yoga is reflected in the outcomes achieved outcomes?

**Figure 5:**
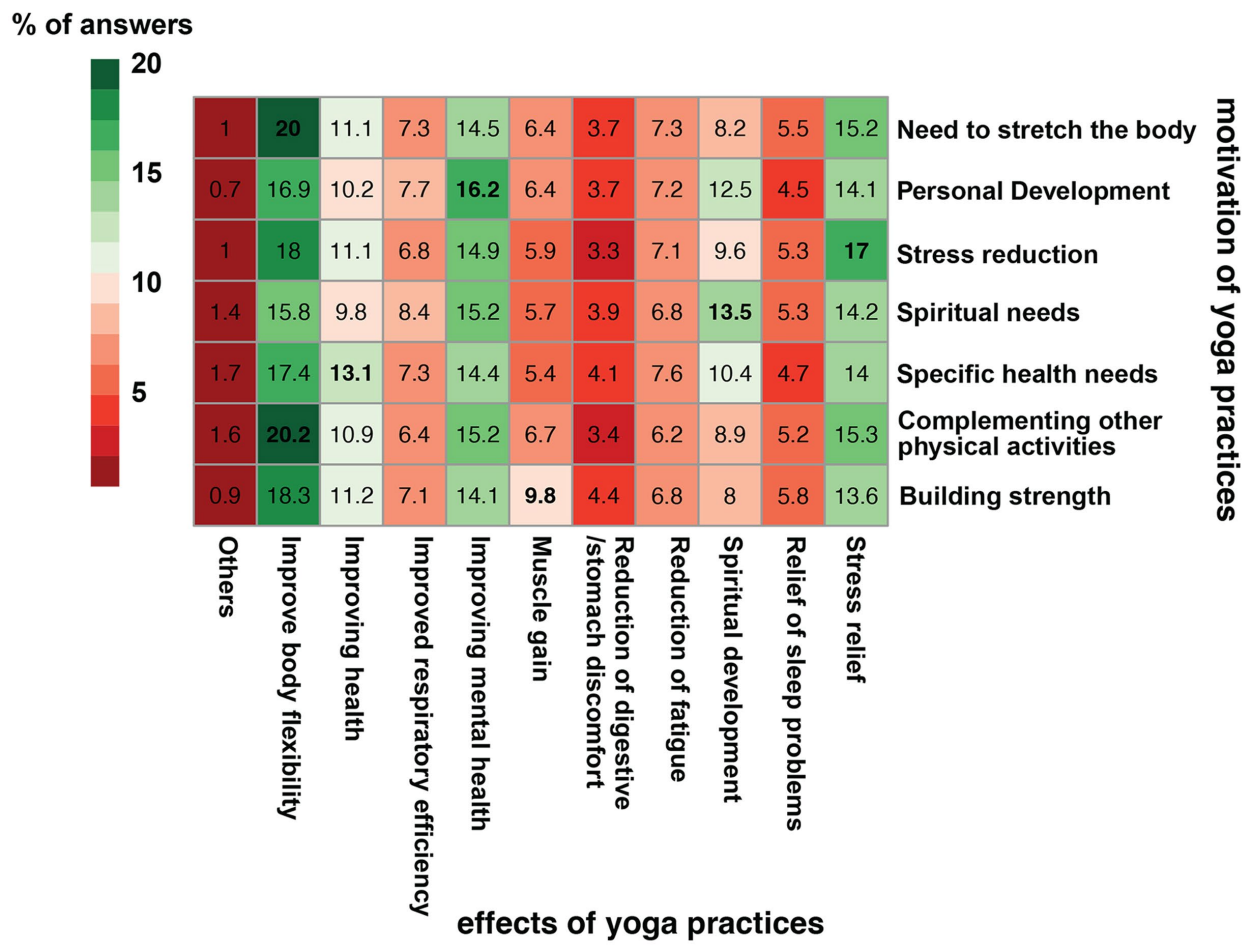
two-way frequency table heatmap showing the frequency dependence of responses to individual motivations for yoga practice in relation to the effects of yoga practice. The data in the heatmap shows the percentage distribution of responses to the motivation question.

In order to answer this question, the data from the question on motivation and the effects of yoga practice were analyzed as a two-way frequency table in Figure 5. The percentage distribution of the individual response from the question on motivation in relation to the effects of yoga practice is presented as a heatmap. The effect of yoga practice as improving body flexibility was the most frequently selected in each group however, the motivations for undertaking yoga practice overlapped with the achieved effects of yoga practice. Those whose motivation was stress reduction most often indicated that the effect of yoga practice was stress relief (17%). Those motivated by the need to stretch the body indicated improving body flexibility as an effect of the practice.

## Discussion

Although the causes of occupational burnout remain unclear, in the last few decades, there has been much interest in occupational stress in health sciences (49–52). Research shows that professional work is increasingly becoming a cause of chronic stress, which can lead to occupational burnout and consequently to deterioration of health, quality of life and the development of such diseases and disorders as depression, PTSD, anxiety, musculoskeletal disorders, cardiovascular diseases; and the development of maladaptive behavior. Through the release of stress hormones, induction of inflammation, and suppression of immunity, chronic stress can encourage tumorigenesis and oncogenesis (tumor formation). In addition, chronic stress causes the neuroendocrine system (hypothalamus-pituitary–adrenal axis) and sympathetic nervous system to become activated, producing stress hormones that can promote tumor growth and control the tumor microenvironment (53). Chronic stress and high glucocorticoid levels alter the brain’s structure and function, particularly the hippocampus, a crucial component of the limbic system that is essential for cognitive processes like learning and remembering (54).

According to the World Health Organization (WHO), occupational burnout is a syndrome resulting from chronic work-related stress, with symptoms characterized by “feelings of energy depletion or exhaustion; increased mental distance from one’s job, or feelings of negativism or cynicism related to one’s job; and reduced professional efficacy”^1^.

Although the ICD-11 classification does not include occupational stress, it can be classified as a 6B4Y, i.e., other specified disorders specifically related to stress. However, it should be remembered that occupational burnout is a consequence of long-term work-related stress. The most common causes of work-related stress include routine, uncomfortable working conditions, long working hours, competitive, conflicted work environment, pressure, control and lack of trust from the employer, unstable employment conditions, and mobbing. Chronic stress that causes professional burnout can have negative effects, not only at the individual level, e.g., deterioration of health and quality of work, but also at the organizational and social level (55).

Our research showed that in the group of people predisposed by age to work, the dominant factor in deciding to start yoga practice was the desire to reduce stress (Figure 1). It should be noted that the upward trend of this factor appears already in the group of people aged 25–30, i.e., when most people start their first professional job. This tendency significantly intensifies in people over 30, indicating the first signs of occupational stress. This trend continues throughout the entire period of active professional work. Attention is drawn to the fact that stress reduction is no longer the key motivating goal for people entitled to retirement. It is rare for individuals with age-related pension entitlements to begin a yoga practice due to stress, which supports the thesis that work is the primary cause of stress for individuals between the ages of 25 and 60. Jarvelin-Pasanen et al. have previously suggested that the criterion of working age predisposes individuals to occupational stress (56). Our research results align with the conclusions of Shoman et al. and Maslash, who conclude that work stress is positively related to occupational burnout and reinforces the concept that occupational burnout is a response to excessive stress at work (51). Researchers dealing with the problem of occupational stress of employees in the health care system are coming to similar conclusions (57–59). Chen et al. have shown that burnout facilitates the relationship between occupational stress and symptoms of depression and anxiety in young nurses (60).

Yoga is one of the forms of mindfulness techniques, both in its dynamic form (focusing on movement and breathing) and in the form of relaxation techniques. The regular practice of mindfulness is, among other things, linked to the functioning of various areas of the brain, including the anterior cingulate cortex (ACC). People who meditate regularly show greater activity in the ACC area, which is responsible for self-regulation and drawing conclusions based on experience, thereby aiding optimal decision-making (has an essential role in both learning and using extended action-outcome histories to optimize voluntary choice behavior) (17, 60).

It also comprises such stress-reducing methods as stretching techniques and social support (61). In research on the effectiveness of mindfulness interventions, Kimberly notes that online mindfulness interventions seem practical and effective in decreasing employee stress while improving resiliency, vigor, and work engagement, thereby enhancing overall employee well-being (62). Green and Kinchen came to similar conclusions, indicating that mindfulness training prevents professional burnout in nurses and helps to deal with difficult situations (63). Reduced burnout, perceived stress, and increased mindfulness were demonstrated in a study on a group participating in a four-hour mindfulness workshop (64). Cabat-Zinn observed that mindfulness-based stress reduction (MBSR) might cause brain alterations that make it easier to deal with unpleasant emotions when under stress. These modifications persisted for at least 4 months following the operation. Yoga practice does not always have to be associated with spiritual aspects, which was confirmed by our respondents (Figure 3). Yoga and physical therapy help reduce perceived stress in low-income persons with chronic low back pain (cLBP), according to Berlowirtz et al. Moreover, physical exercise was more efficient than educational intervention (65). The study (a systematic review and metanalysis) by Wand et al. found that yoga interventions can help manage sleep problems in women when compared to non-active control conditions (60).

A meta-analysis by Pascoe et al. indicates that the physical practice of yoga can positively impact human well-being. As a result of the study, they also claim that yoga asanas are associated with improved regulation of the sympathetic nervous system and hypothalamic–pituitary–adrenal system in various populations (36, 66, 67). This study also showed that yoga significantly reduces cortisol measured in saliva during wakefulness and sleep, which is extremely important because long-term elevated cortisol levels are a predisposing factor for depression (68, 69). Cortisol levels can be lowered with properly planned physical activity (70–72), whereas intense exercise raises stress hormone levels (36). In the analysis of Pascoe et al., they make no distinction in terms of styles of yoga practice. Nevertheless, every yoga practice begins with calming the breath (pranayama) and ends with relaxation. All yoga styles also include mindfulness elements in their technique, such as focusing on the breath, body scanning or other forms of mindfulness techniques.

Among all yoga styles, our respondents most often chose vinyasa yoga, and their decision was motivated by the desire to reduce stress levels. However, our respondents indicated that in yoga, they value the combination of the physical and spiritual aspects, which mindfulness techniques can establish. Significantly few people indicated that spiritual motivation is the main reason for exercise (Figure 4). Vinyasa yoga is a dynamic style characterized by the diversity that allows practitioners to choose the intensity of the practice according to their needs and abilities (37, 73, 74). The impact of vinyasa yoga practice on reducing stress and reducing problems with falling asleep has already been shown in a study on a group of oncologists who declared an improvement in well-being after 3 months of practice (37). Notably, in clusters 4 and 5 (Figure 3), the respondents to whom the desire to reduce stress through yoga practice was assigned were predominantly those with a short history of the practice allowing us to assume, as confirmed by the results of other researchers, that yoga has a positive effect on stress management role (67, 75, 76). Respondents who have been practicing for years are less likely to report a need for stress reduction (Figure 4). Balakrishnan et al. link this to a better-developed parasympathetic nervous system in yogis. The parasympathetic predominance demonstrated in the yoga group suggests that hatha yoga practitioners may be at a lower risk of stress-related comorbidities (77). Akdeniz concludes his research with the recommendation that yoga should be started at a young age and practiced regularly, as it provides a simple solution to significant problems such as pain, anxiety, sleep and stress that negatively affect our daily lives (41).

It is worth noting that the respondents achieved the expected results through yoga practice, as presented in Figure 4.

It should be noted that chronic stress has been widely described as a cause of many severe diseases and disorders, including cardiovascular diseases (78–82). The impact of physical activity on well-being and chronic stress is increasingly described in literature (84–87); its importance was noticed both in the group of healthy people and those struggling with diseases (83–86). Yoga combines physical, breathing, and mindfulness exercises and may benefit HF patients and those struggling with the affective disorder (33, 87, 88).

Research results have shown that yoga practice significantly reduces physiological stress and inflammation, improves well-being and sleep quality, and practice is safe, meaning it can be used not only as a prophylaxis but also as a support to conventional treatment of diseases caused by occupational stress.

## Conclusion

Occupational stress contributes to many diseases and psycho-somatic disorders. Yoga practice can effectively neutralize occupational stress.

Yoga combines the benefits of physical activity and mindfulness, making it possible to take care of both physical and mental health. Yoga practice is an inexpensive and side-effect-free way to improve human well-being. However, research should be conducted to understand the mechanisms of the exercises.

### Limitations

While acknowledging that work-related stress is a significant issue in modern society, it’s imperative to recognize the limitations of this study. Notably, despite the widespread use of the Internet and social media among working individuals, it’s crucial to bear in mind that not everyone maintains accounts on platforms like Facebook or Instagram. Additionally, a limitation arises from the underrepresentation of individuals of retirement age, who typically have minimal engagement with online resources.

Another aspect to consider is the deliberate use of a custom survey form. This choice was made due to prior research indicating the positive impact of yoga on enhancing life quality and managing stress-related effects. Our specific aim was to scrutinize the intricate correlation between various yoga practices, motivational factors, exercise effects, and the age demographics of the participants. This depth of analysis wasn’t feasible using existing standardized methods.

We acknowledge the inherent constraints associated with this survey format. Nevertheless, our objective was to design a form that could accurately align the type of yoga practice with the practitioners’ specific needs. Our intent is to tailor future assessments more precisely to better cater to the requirements of yoga practitioners.

## Data availability statement

The original contributions presented in the study are included in the article/Supplementary material, further inquiries can be directed to the corresponding author.

## Ethics statement

The studies involving humans were approved by Komisja Bioetyczna przy Uniwersytecie Medycznym im. Karola Marcinkowskiego w Poznaniu ul. Bukowska 70, pok. A204 60–812 Poznań. The studies were conducted in accordance with the local legislation and institutional requirements. The ethics committee/institutional review board waived the requirement of written informed consent for participation from the participants or the participants’ legal guardians/next of kin because Participants completed the questionnaire using an online form, which made it impossible to sign the consent in person. The consent form was attached to the questionnaire, and participants were informed that completing the questionnaire meant participating in the study. In addition, participants had to confirm their consent by selecting the appropriate answer.

## Author contributions

AZ: Conceptualization, Data curation, Formal analysis, Methodology, Project administration, Resources, Visualization, Writing – original draft, Writing – review & editing. MM: Formal analysis, Resources, Writing – review & editing. AB: Data curation, Writing – review & editing. MC: Data curation, Writing – review & editing.

## Footnotes

^1^Burnout an “occupational phenomenon”: International Classification of Diseases. WHO. 28 May 2019. Retrieved 2019-06-01.
